# A Paintable Small-Molecule Hydrogel with Antimicrobial and ROS Scavenging Activities for Burn Wound Healing

**DOI:** 10.3390/gels10100621

**Published:** 2024-09-26

**Authors:** Qingchun Ji, Kehan Chen, Han Yi, Bingfang He, Tianyue Jiang

**Affiliations:** School of Pharmaceutical Sciences, Nanjing Tech University, Nanjing 211816, China

**Keywords:** small-molecular hydrogel, burn wound, caffeic acid, antimicrobial, ROS scavenging

## Abstract

Delayed wound healing induced by bacterial infection and a persistent inflammatory response remains a great clinical challenge. Herein, we reported a paintable, anti-bacterial, and anti-inflammatory Nap-F3K-CA (Nap-Phe-Phe-Phe-Lys-Caffeic Acid) hydrogel for burn wound management based on caffeic acid (CA)-functionalized short peptides (Nap-Phe-Phe-Phe-Lys). Hydrogels are assembled by non-covalent interactions between gelators, and the incorporation of CA promotes the self-assembly of the hydrogel. After being applied to burn wounds, the hydrogel effectively adapted to irregular wound beds and maintained a moist protective environment at the wound. The Nap-F3K-CA hydrogel can scavenge ROS to relieve oxidative damage and downregulate proinflammatory levels. The Nap-F3K-CA hydrogel also displayed potent antibacterial activity against Gram-positive and Gram-negative bacteria, which reduced the incidence of wound infections. Moreover, the hydrogel exhibited good biocompatibility and hemostatic function. In vivo experiments demonstrated that the Nap-F3K-CA hydrogel significantly accelerated the repair of the skin structure including promoting collagen deposition, vascular regeneration, and hair follicle formation. These findings proved the clinical application potential of the Nap-F3K-CA hydrogel as a promising burn wound dressing.

## 1. Introduction

Burn wound healing is a long-term process involving hemostasis, inflammation, proliferation, and remodeling [1,2,3]. The inflammatory phase refers to a phase of the massive infiltration of a variety of pro-inflammatory cells, such as macrophages and neutrophils, the induction of the release of a variety of inflammatory mediators, and the generation of large quantities of reactive oxygen species (ROS), after the onset of a burn injury [1,4,5]. However, the invasion of microorganisms such as bacteria and wound infection caused by them can lead to an uncontrolled increase of pro-inflammatory cytokines and ROS, promoting the metabolic reprogramming of macrophages, and thus deteriorating the inflammatory circulation [6,7,8]. This prolonged high level of inflammation can cause the wound to enter a chronic phase, making it difficult to heal [9]. Therefore, effective free radical scavenging, preventing bacterial infection, and controlling inflammation deterioration are particularly important in burn wound management.

Hydrogels have gained much attention in the biomedical field due to their softness, high water content, pore structure, and good biosafety [10,11,12]. With physical barrier and moisturizing functions, it can provide wound protection and promote wound healing [13]. A hydrogel incorporating antioxidants shows the effective alleviation of inflammation in the wound healing. Chen and coworkers [14] photo-crosslinked the antioxidant epigallocatechin-3-gallate (EGCG) with a 4-carboxyphenyboronic acid (CPBA) modified gelatin methacryloyl (GelMA) hydrogel to prepare a GMPE (GelMA-CPBA/EGCG) hydrogel which was used for ROS scavenging and inflammation relief in diabetic wounds. Xiao and coworkers [15] successfully prepared an antioxidant citrate-based hydrogel and demonstrated that it could achieve a slow release of copper ions while maintaining antioxidant properties to promote chronic wound healing. Liu and coworkers [16] added the antioxidant tannic acid to polyvinyl alcohol and agarose-based hydrogels for accelerating wound healing and inhibiting scarring. Small-molecule hydrogels that are formed by the self-assembly of small molecules open an avenue for burn wound management [17,18,19,20]. They can effectively adapt to irregular wound beds because gelators are assembled into nanofibers based on non-covalent interactions [21,22]. There is a great demand for the development of multifunctional wound-healing dressings based on small-molecule hydrogels for the complex burn wound microenvironment.

Here, we reported a paintable small-molecule hydrogel with antimicrobial and ROS scavenging activities for burn wound healing. Caffeic acid (CA) is a naturally occurring polyphenolic compound that contains catechol and acrylic acid functional groups in its structure [23,24,25]. It is reported that CA shows favorable antioxidant and free radical scavenging activity [26,27], along with a wide range of anti-inflammatory [28] and antimicrobial effects [29]. We designed a novel multifunctional oligopeptide gel factor, Nap-F3K-CA, by combining caffeic acid with the sequence Nap-F3K (Nap-Phe-Phe-Phe-Lys). Nap-F3K is composed of Phe-Phe-Phe (F3), which is a commonly used building block by promoting π-π stacking in the self-assembling, 2-naphthalene acetic acid (Nap) as the capping group, and lysine (K) as a linking group between the caffeic acid and peptide. The introduction of caffeic acid not only conferred ROS scavenging and antimicrobial efficacy to Nap-F3K-CA but also promoted the self-assembly of the hydrogel driven by non-covalent interactions between gelators, which helped to shorten the hydrogel formation time and improve the mechanical properties of the formed hydrogel. The hydrogel it forms is characterized by good compatibility and degradation, and can effectively adapt to irregular wound beds because of its favorable deformability and injectability.

After its application to burn wounds, the Nap-F3K-CA hydrogel could maintain a moist environment at the wound site, efficiently inhibit infection, and remove excessive reactive oxygen species, thus reducing the inflammatory response. Through the rat burn wound model, we demonstrated that the Nap-F3K-CA hydrogel reduced the expression of pro-inflammatory cytokines at the wound site in rats, accelerated the formation of collagen fibers, promoted the regeneration of the skin tissues and appendages, and improved the wound healing rate. We believe that this study provides a new strategy for burn wounds as well as other forms of chronic wound healing.

## 2. Results and Discussion

### 2.1. Self-Assembly and Characterization of Nap-F3K-CA Hydrogel

**Figure 1:**
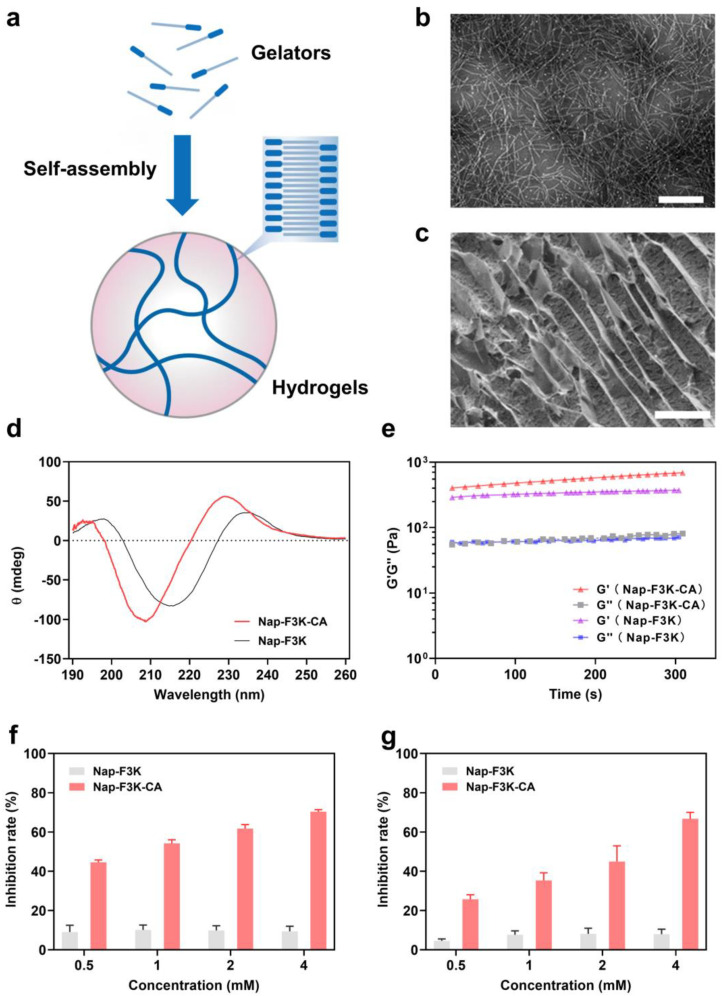
(**a**) Schematic representation of peptide self-assembly into hydrogels. (**b**) TEM image of Nap-F3K-CA hydrogel, scale bar = 200 nm. (**c**) SEM image of Nap-F3K-CA hydrogel, scale bar = 50 μm. (**d**) CD spectrum of Nap-F3K-CA and Nap-F3K hydrogels. (**e**) Rheological measurement in dynamic time scanning mode of Nap-F3K-CA and Nap-F3K hydrogels, G′: storage modulus, G″: loss modulus. DPPH (**f**) and ABTS (**g**) inhibition rate of Nap-F3K-CA and Nap-F3K hydrogels.

The Nap-F3K-CA hydrogel was formed by adjusting the pH from alkaline to neutral. The assembly-into-hydrogels process is shown in Figure 1a. The minimum gelation concentration of Nap-F3K-CA was about 0.25% (*w*/*v*), which was significantly lower than the 1.5% (*w*/*v*) of Nap-F3K. It was speculated that the introduction of caffeic acid facilitates the self-assembly of gelators through π-π stacking to form hydrogels. The transmission electron microscope (TEM) images showed that Nap-F3K-CA can assemble into long nanofibers with a diameter of 7 nm (Figure 1b), while a short nanofiber structure was observed in the Nap-F3K sample (Figure S1). It was speculated that the long nanofiber of Nap-F3K-CA is conducive to gelling water and forming a hydrogel. Scanning electron microscopy (SEM) showed a porous network structure of the Nap-F3K-CA hydrogel (Figure 1c). Brunauer–Emmett–Teller (BET) analysis was applied to assessed the surface area and pore size distribution of the Nap-F3K-CA hydrogels by nitrogen adsorption on the surface of the sample under constant temperature conditions. The surface area and pore volume of the Nap-F3K-CA hydrogel was 16.55 m^2^/g and 0.006 cm^3^/g, respectively, which further validated the porous structure of the hydrogel.

The circular dichroism (CD) spectra measurement was conducted to analyze the secondary structure of Nap-F3K-CA. As shown in Figure 1d, a strong positive signal near 196 nm and a negative signal near 208 nm indicated that Nap-F3K-CA self-assembles into a β-sheet structure. Next, the mechanical properties of the Nap-F3K-CA hydrogel were detected by the rotational rheometer. The results of the dynamic time mode scan are shown in Figure 1e. The storage modulus (G′) exceeded the loss modulus (G″), which reconfirmed the hydrogel formation. In addition, the Nap-F3K-CA hydrogel exhibited a higher value of G′ compared to the Nap-F3K hydrogel, which may be due to the fact that the introduction of CA endows the hydrogel with a higher mechanical strength.

The 2,2′-azino-bis(3-ethylbenzothiazoline)-6-sulphonic acid (ABTS) radicals and 2,2-diphenyl-1-picrylhydrazyl (DPPH) method can be used to evaluate the antioxidant capacity of the substance. After the tested substance is added to the free radical solution, the antioxidant components can react with the free radical and make the absorbance of 734 and 517 nm decrease, separately. To determine the free radical scavenging capability of the hydrogels, we measured the scavenging efficiency of the Nap-F3K and Nap-F3K-CA hydrogels for both radicals. As shown in Figure 1f,g, compared with the weak scavenging effect of the Nap-F3K hydrogel, Nap-F3K-CA showed a better scavenging efficiency for both free radicals, and the scavenging efficiency increased with increasing concentration. The inhibition of DPPH and ABTS by the 4 mM Nap-F3K-CA hydrogel reached 70.3% and 66.7%, respectively, indicating its favorable antioxidant capacity.

In addition, the UV absorption spectrum variation of the ABTS and DPPH radicals during co-incubation with the Nap-F3K-CA hydrogel was scanned; the Nap-F3K-CA hydrogel treatment led to a rapid decline in the maximum absorption of DPPH at 517 nm and of ABTS at 734 nm, respectively, and presented time-dependent radical scavenging within 60 min (Figure S2).

### 2.2. Biocompatibility Evaluation of Nap-F3K-CA In Vitro

**Figure 2:**
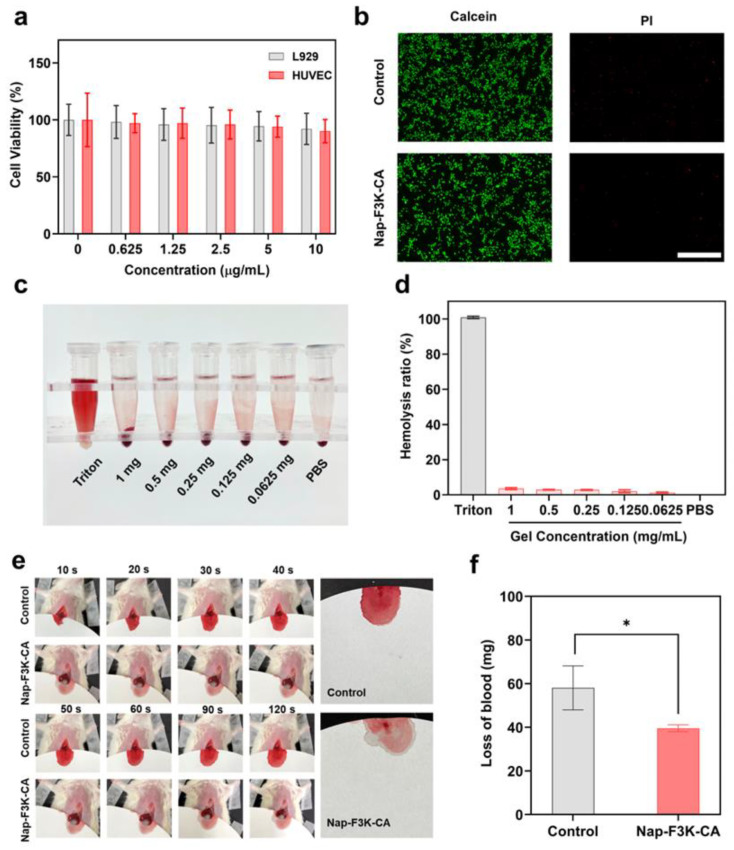
(**a**) Viability of L929 cells and HUVECs after treatment with different concentrations of Nap-F3K-CA hydrogel. (**b**) Calcein-AM/PI fluorescence staining images of HUVECs after incubation with Nap-F3K-CA hydrogel, scale bar = 500 μm. (**c**) Hemolysis test of Nap-F3K-CA hydrogels with different concentrations. (**d**) Hemolysis ratio of different formulations. (**e**) The bleeding images of the mouse liver hemorrhage model. (**f**) Quantitative analysis of loss of blood, * *p* < 0.05.

The excellent biocompatibility of hydrogels is a prerequisite for their use as wound dressings [30]. We first evaluated the cytotoxicity of the Nap-F3K-CA hydrogel by the MTT ((3-(4,5-dimethylthiazol-2-yl)-2,5-diphenyltetrazolium bromide) tetrazolium assay. As displayed in Figure 2a, the cell viabilities of NCTC clone 929 (L929) cells and human umbilical vein endothelial cells (HUVECs) co-incubated with various concentrations of the Nap-F3K-CA hydrogel were both higher than 90%, which suggested that the Nap-F3K-CA hydrogel exhibits negligible toxicity towards L929 cells and HUVECs. In addition, the HUVECs incubated with the Nap-F3K-CA hydrogel were stained by a live/dead staining kit. The green fluorescence of calcein acetoxymethyl ester (Calcein-AM) can stain living cells, while propidium iodide (PI) dye with red fluorescence only passes through dead cells. As shown in Figure 2b, after treatment with the Nap-F3K-CA hydrogel, HUVECs showed strong green fluorescence, and almost no red fluorescence was observed, indicating that the cells continue to have a high viability. The above results showed the good cytocompatibility of the Nap-F3K-CA hydrogels.

The in vitro hemolysis test was performed by incubating red blood cells with the Nap-F3K-CA hydrogel over time. The absorbance of hemoglobin in the supernatant after centrifugation was detected (Figure 2c,d). In contrast to the strong hemolytic symptom of the Triton-X-100-treated group, the absorbance of the saline and Nap-F3K-CA hydrogel group was comparably low, indicating the Nap-F3K-CA hydrogel does not cause hemolysis. Hemostasis is an important step in the treatment of trauma, and studies have shown that phenolics such as caffeic acid can interact with nucleophilic reagents in blood proteins to accelerate platelet recruitment and coagulation factor activation [31]. The acute hemostatic performance of the hydrogels was assessed by a mouse liver incision model (Figure 2e). The quantitative analysis of blood loss is shown in Figure 2f. The amount of bleeding in the hydrogel group was 39.6 mg, while the bleeding amount in the control group was 58.1 mg. The amount of bleeding in the Nap-F3K-CA hydrogel group was reduced by 32% in comparison with the control group, which indicated a potent hemostatic ability of the Nap-F3K-CA hydrogel, which could provide positive effects in the early stage of wound healing.

### 2.3. Anti-Infection Evaluation

**Figure 3:**
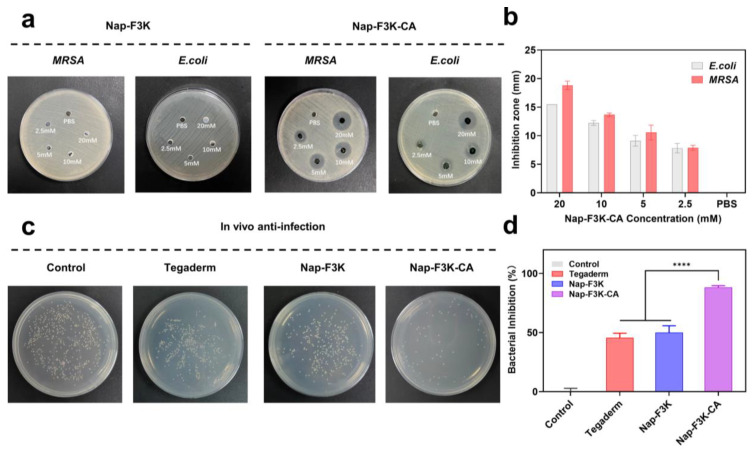
(**a**) Inhibition zone and quantitative analysis (**b**) of different concentrations of Nap-F3K-CA hydrogel against *MRSA* and *E. coli.* (**c**) In vivo antibacterial properties of Nap-F3K-CA hydrogel: images of bacterial colonies and (**d**) bacterial inhibition percentage, **** *p* < 0.0001.

Open wounds are often accompanied by secondary bacterial infections, which may lead to delayed wound healing and complications [32]. To test the antibacterial capability of the Nap-F3K-CA hydrogel, Methicillin-resistant *Staphylococcus aureus* (*MRSA*), and *Escherichia coli* (*E. coli*) were cultured with the hydrogels at different concentrations. As shown in Figure 3a,b, the inhibition zones of varying diameters were formed around the Nap-F3K-CA hydrogels at different concentrations, and the diameters increased with the increase in concentration, in which the 20 mM Nap-F3K-CA hydrogel formed inhibition zones of about 1.5 cm and 1.8 cm in *E. coli* and *MRSA*, respectively. By contrast, no obvious inhibition zone was formed around the Nap-F3K hydrogel, indicating that CA at the C-terminus endows the hydrogel with antibacterial properties.

Furthermore, we assessed the in vivo antibacterial capability of Nap-F3K-CA against *MRSA* by an infected burn wound model. The Tegaderm hydrogel is a sterile and amorphous hydrogel formulated to provide a moist environment for a wound, which promotes wound healing and prevents wound desiccation. It has been widely used as a control in research in the field of wound healing [33,34]. Herein, we also used it as a comparison for the Nap-F3K and Nap-F3K-CA hydrogels. As displayed in Figure 3c, compared to the control group, a great inhibition on *MRSA* was observed in the Nap-F3K-CA hydrogel treatment group with only a small number of bacterial colonies observed. The sterilization ratio of the Nap-F3K-CA hydrogel reached 88.17%, significantly higher than the commercial Tegaderm hydrogel group of 43.5% and the Nap-F3K hydrogel group of 50.0% (Figure 3d), suggesting that the Nap-F3K-CA hydrogel had favorable antimicrobial activity. This would help to prevent bacterial infection at the wound site, avoiding the effects of bacteria and other microorganisms on the repair and regeneration of the skin tissue at the wound site.

### 2.4. Effects of Nap-F3K-CA Hydrogel on Burn Wound Healing

**Figure 4:**
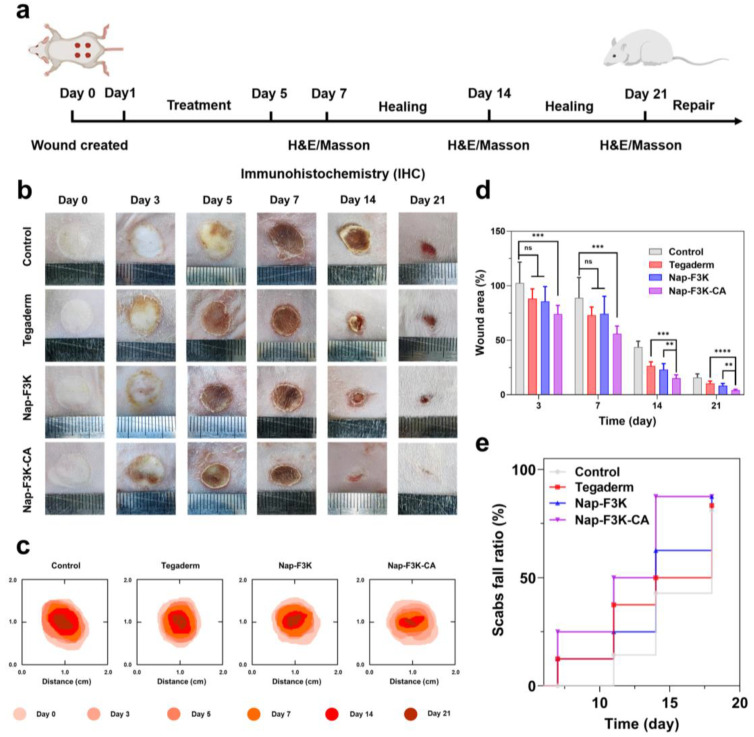
(**a**) Establishment of rat burn model and treatment process. (**b**) Burn wound images of different treatment groups within 21 days. (**c**) A model of burn wound changes. (**d**) Quantification of wound area, ** *p* < 0.01, *** *p* < 0.001, **** *p* < 0.0001. ns = not significant. (**e**) Rat scab removal rate.

Having demonstrated the ROS scavenging and antimicrobial capacity, as well as the good biosafety of the Nap-F3K-CA hydrogel, we further investigated the burn-wound-healing-promoting capability of the Nap-F3K-CA hydrogel on the rat burn model. The treatment regimen is illustrated in Figure 4a.

As shown in Figure 4b–d, burn wounds in the different treatment groups gradually healed over time. After 3 days of modeling, all wounds were accompanied by slight edema. On Day 5, the wound area of each hydrogel treatment group exhibited a slight decrease and was accompanied by the formation of scabs, whereas scabs appeared on Day 7 in the control group. In addition, on Day 7, the Nap-F3K-CA hydrogel reduced the wound area to 55.93% of the original, notably lower than the control group of 88.75%. The wound area of each group was further reduced with the extension of treatment time. In the control group, there were still scabs attached to the wound, while, in the Nap-F3K-CA hydrogel group, the scabs gradually fell off from Day 7 to Day 14, and completely fell off on Day 14. This phenomenon is consistent with the wound healing process, in which scabs first develop, then the scabs fall off and the wound begins to recover [35,36]. The Nap-F3K hydrogel and Tegaderm accelerated the recovery of burn wounds to some extent, which was probably ascribed to the moist environment provided by the hydrogel to promote wound healing [37,38,39]. On Day 21, the healing rate of the Nap-F3K-CA hydrogel group reached 95.86%. Throughout the whole healing process, treatment with the Nap-F3K-CA hydrogel resulted in a notable acceleration in the healing rate of the wound, and the wound area was significantly lower than the other hydrogel treatment groups in the later stages of treatment. Meanwhile, we recorded the time of scab removal in different groups. As shown in Figure 4e, the scab fall ratio of the Nap-F3K-CA-hydrogel-treated wounds was 87.5% on Day 14, while the wounds in the control group had a ratio of only 37.5%, which demonstrated the ability of the Nap-F3K-CA hydrogel to promote wound healing. This is consistent with the wound healing process, in which scabs first develop, then the scabs fall off and the wound begins to recover.

### 2.5. Histological and Immunochemical Analysis

**Figure 5:**
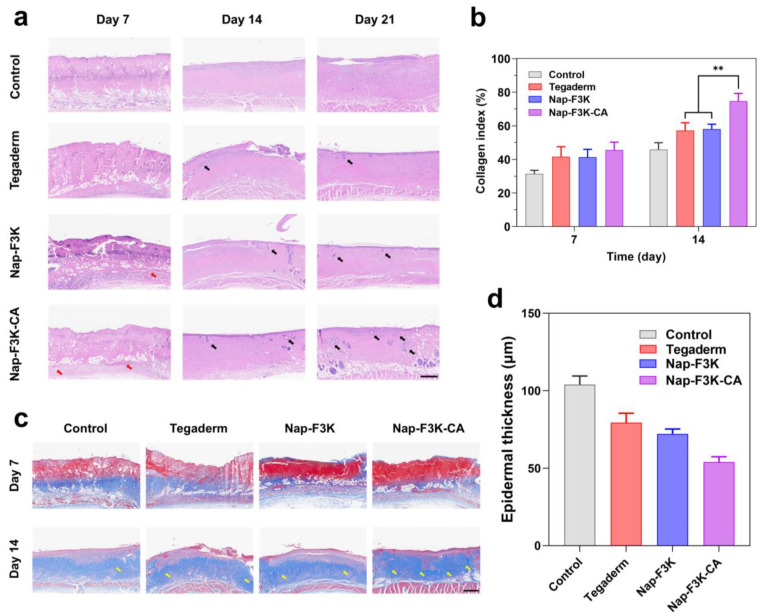
(**a**) H&E-stained skin tissues of rats in different groups: black arrows indicate hair follicles and sebaceous glands, and red arrows indicate blood vessels scale bar = 500 μm. (**b**) Masson-stained skin tissues of rats in different groups: yellow arrows indicate collagen fibres, scale bar = 500 μm; ** *p* < 0.01. (**c**) Collagen index quantification assay. (**d**) Rat epidermal thickness quantification assay.

We further used hematoxylin–eosin (H&E) and Masson trichrome staining to assess the skin structure, appendage recovery, and collagen deposition during wound healing. The H&E-stained images (Figure 5a) on Day 7 showed that the wound site of the control group appeared to have an incomplete skin structure and a loose dermal matrix. In addition, the junction of the necrotic tissue and wound was infiltrated with a large number of inflammatory cells. By contrast, the Nap-F3K-CA hydrogel group had less inflammatory infiltration of the wound site, along with a small amount of new granulation tissue and capillaries. On Day 14, the necrotic tissues of the Nap-F3K hydrogel and Tegaderm group were shed, and the granulation tissue increased significantly, while the epithelial structure of the Nap-F3K-CA group had been restored, and new hair follicles and sebaceous glands appeared (black arrow). After 21 days, the Nap-F3K-CA-treated wounds had regained their skin tissue structure and were closest to normal skin.

The results of Masson trichrome staining revealed that the collagen fibers (yellow arrows) distributed in the dermis in the control group and other treatment groups were less stained and arranged sparsely, while the number of collagen fibers distributed in the dermis in the Nap-F3K-CA hydrogel group was greater and they were arranged evenly (Figure 5b). The collagen index is an important indicator for evaluating collagen deposition, with higher values representing better collagen deposition in the wound site. The results showed that the collagen index was much higher in the Nap-F3K-CA hydrogel treatment group than in the other treatment groups (Figure 5c). In addition, the epidermal thickness of rats after Nap-F3K-CA hydrogel treatment was minimal, indicating that it promotes the recovery of the skin structure (Figure 5d). The results of H&E and Masson staining showed that the Nap-F3K-CA hydrogel could enhance epidermal growth, increase collagen deposition, and promote wound healing.

**Figure 6:**
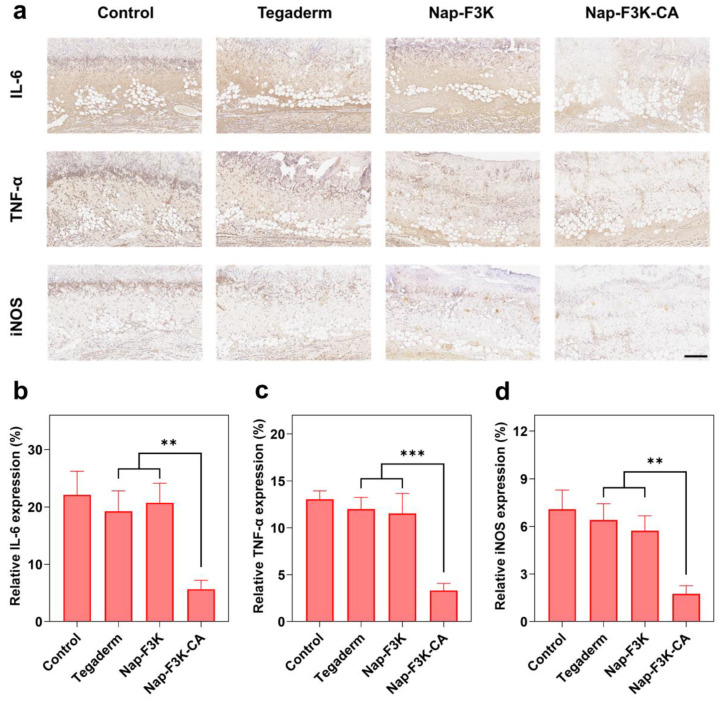
(**a**) Immunohistochemical staining images of skin in each group of rats on Day 7, scale bar = 200 μm. Immunohistochemical analysis of IL-6 (**b**), TNF-α (**c**), and iNOS (**d**); ** *p* < 0.01, *** *p* < 0.001.

Inflammation occurs at 24 h after burn injury and is characterized by the release of various inflammatory mediators [1]. However, a prolonged inflammatory state can impede the process of burn wound recovery. To further confirm the inflammation regulation effect of hydrogels, immunohistochemical staining of tumor necrosis factor-α (TNF-α), IL-6, and iNOS in the skin tissues was performed. As shown in Figure 6a, all groups showed different degrees of inflammatory response on Day 7. The control group exhibited a high expression of TNF-α, IL-6, and iNOS. In contrast, the Nap-F3K-CA hydrogel treatment notably downregulated the inflammatory levels at the wounds by providing antibacterial protection and reducing ROS production. The relative expression of the three inflammatory factors in the Nap-F3K-CA hydrogel group was only 3.33%, 5.67%, and 1.75%, significantly lower than other treatment groups (Figure 6b–d). In conclusion, the Nap-F3K-CA hydrogel showed excellent anti-inflammatory, antibacterial, and pro-repair functions, promoted collagen deposition and skin tissue recovery, and, to a certain extent, demonstrated its superiority over commercially available hydrogel dressings.

## 3. Conclusions

In this work, a paintable oligopeptide hydrogel Nap-F3K-CA based on natural polyphenol-caffeic acid was developed for burn wound management. Its favorable mechanical properties and paintability make it suitable for all kinds of irregular wounds. In addition, the introduction of caffeic acid improves the self-assembly performance of the gelators, endows the hydrogel with antibacterial, anti-inflammatory, and antioxidant properties, and maintains good biocompatibility. The experiments proved that the Nap-F3K-CA hydrogel can effectively prevent the invasion of bacteria into the wound, inhibit inflammation, induce angiogenesis, and increase collagen deposition at the wound site after being applied, thus promoting the regeneration of the skin structure and wound repair. Therefore, the developed hydrogel dressing can offer a promising solution for chronic wound management as well as the regeneration of various damaged tissues.

## 4. Materials and Methods

### 4.1. Materials

Nap-F3K-CA and Nap-F3K peptides were obtained from GL Biochem Co., Ltd. (Shanghai, China). 2,2-diphenyl-1-picrylhydrazyl (DPPH) and 2,2′-azino-bis(3-ethylbenzothiazoline)-6-sulphonic acid (ABTS) free radical scavenging capacity assay kits were purchased from Solarbio (Beijing, China). Calcein-AM/PI staining was provided by Beyotime (Shanghai, China). Tegaderm hydrogel was obtained from 3M Company (St. Paul, MN, USA).

### 4.2. Preparation and Characterisation of Nap-F3K and Nap-F3K-CA Hydrogels

Equimolar amounts of Nap-F3K and Nap-F3K-CA lyophilized powders were weighed and dissolved in distilled water, and NaOH (1 M) was added to the solution until the lyophilized powders were completely dissolved, and then HCl (0.4 M) was added to the peptide solution to pH 7.3. The hydrogel was formed after 2 min at room temperature. The final concentration of hydrogel was 20 mM. The morphology of Nap-F3K and Nap-F3K-CA hydrogels were characterized by TEM (Hitachi H7800, Ibaraki, Japan) and SEM (Hitachi Regulus-8100, Ibaraki, Japan). The JWGB-BK200B BET analyser (Beijing, China) was used for evaluating the surface area and pore volume of hydrogel through gas adsorption method.

### 4.3. CD Measurement

The Nap-F3K-CA hydrogels were diluted with deionized water to 0.2 mg/mL and measured by CD spectroscopy (JASCO J-1500, Tokyo, Japan). The scanning range was set at 190–260 nm with an interval of 50 nm.

### 4.4. Rheological Analysis

The 500 μL hydrogel samples were loaded onto the rheometer (Anton Paar MCR 302, Graz, Austria) platform using a syringe. Then, the measurements were performed at 2% strain amplitude and 2 Hz frequency. The storage modulus and loss modulus were recorded.

### 4.5. Free Radical Scavenging Assay

DPPH radical test solution and ABTS radical test solution were prepared according to the kit instructions. The hydrogel was diluted with water to concentration of 4, 2, 1, and 0.5 mM samples and incubated in the dark at room temperature with the addition of DPPH and ABTS test solution for 30 min. Then, their absorbance values were detected at 517 nm and 734 nm using a UV spectrometer (Hanon i6, Jinan, China), separately.

Then, 200 μL of Nap-F3K or Nap-F3K-CA hydrogel dispersed solutions (4 mM) were added to 2 mL of the above radical test solutions. After predetermined time points, the absorption spectra were measured using a UV spectrometer.

### 4.6. MTT Assay

Mouse fibroblasts (L929) and human umbilical vein endothelial cells (HUVECs) were seeded in 96-well plates and cultured for 24 h, respectively. Subsequently, the cells were co-incubated with different concentrations of hydrogel dilution and incubated for 24 h. Then, 20 μL of MTT was added to each well. After another 4 h of incubation, the supernatant was discarded and 150 μL of dimethyl sulfoxide (DMSO) was added. The absorbance at 570 nm was detected. Finally, the cell viability rate was calculated:

Cell viability rate (%) = (OD_hydrogel_ − OD_blank_)/(OD_control_ − OD_blank_) × 100% (1)

### 4.7. Calcein-AM/PI Live/Dead Staining Assay

HUVECs were seeded in 6-well plates and cultured for 24 h. Subsequently, the cells were incubated with hydrogel dilution solution for 24 h. After phosphate-buffered saline (PBS) washing, the cells were stained by Calcein-AM/PI staining kit, and visualized through an inverted fluorescence microscope imaging system (Nikon Eclipse Ti2, Tokyo, Japan).

### 4.8. Hemolysis Assay

The red blood cells collected from mice were diluted with PBS to prepare a red blood cell suspension (2%, *v*/*v*). Next, the red blood cell suspension was incubated with the hydrogels at different concentrations (0.0625, 0.25, 0.5, and 1 mg/mL) at a ratio of 9:1. After incubation at 37 °C for 6 h, the supernatant was obtained by centrifugation, and its absorbance at 570 nm was detected by a microplate reader.

### 4.9. Hemostasis Test

The anesthetized mice were fixed on the surgical plate. After removing the abdominal hair of each mouse, the liver was exposed and the pre-weighed filter paper was placed under the liver. Liver hemorrhage was induced by a 26 G needle. Immediately, 100 μL of 20 mM Nap-F3K-CA hydrogel was applied to the bleeding site. After 2 min, the blood-absorbing filter paper was weighted and compared with the control group receiving non-treatment.

### 4.10. Antimicrobial Test

The antimicrobial testing was carried out following the standardized protocols detailed in the EUCAST guidelines. *MRSA* or *E. coli* were cultured in LB broth overnight and then spread evenly on LB agar plates (1 × 10^8^ CFU/mL). After punching holes with 4 mm diameter in the agar, the hole is filled with 50 µL of PBS or hydrogel samples at different concentrations (2.5, 5, 10, and 20 mM). After 12 h incubation, the inhibition zones around each sample were measured.

### 4.11. Burn Wound Model Establishment

Female SD rats (~200 g) were provided from the Medicine Center of Yangzhou University. The animal study protocol was approved by the Ethics Committee of Nanjing Tech University (protocol code WX24-56) for studies involving animals. The rats were anesthetized, and the back hair of the rats was removed by hair removal cream. The back was disinfected with 75% ethanol solution. The solid aluminum rod (d = 10 mm, 54 g) was heated to 100 °C with boiling water, and applied to the back of the rat for 15 s under the gravity of the rod, resulting in a total of 4 wounds on the back of the rat. To avoid interactions between the wounds, the distance between the wounds should be above 1 cm.

### 4.12. In Vivo Antibacterial Activity

On Day 1 after the establishment of the rat burn wound model, the scabs on the back of the rats were removed and 10 μL of 10^8^ CFU/mL *MRSA* was seeded to the wound of the rat. Subsequently, different formulations (Tegaderm, Nap-F3K hydrogel, and Nap-F3K-CA hydrogel) were applied to the wound site once a day. After 3 days of treatment, wound secretions were collected and inoculated into LB agar plates. After incubation at 37 °C for 16–18 h, the colony-forming units were counted on the agar plates.

### 4.13. In Vivo Burn Wound Healing Evaluation

Different formulations (Tegaderm, Nap-F3K hydrogel, and Nap-F3K-CA hydrogel) were applied to the wound site once a day for 5 consecutive days, respectively. Wound morphology was photographed and the wound area was recorded at predetermined time points (Day 0, 3, 5, 7, 14, and 21). ImageJ software (ImageJ 1.53c) was used for visualization and analysis of wound healing. The wound area ratio (%) was calculated as the following formula:

Wound area ratio (%) = W_n_∕W_0_ × 100%, (2)

where W_0_ and W_n_ represent the wound area on Day 0 and Day n, respectively.

### 4.14. Histology Staining

During the treatment, at predetermined time points, part of the mice was sacrificed to collect the skin of the wound site and prepared for the H&E staining, Masson trichrome staining, and immunohistochemical staining of TNF-α, IL-6, and iNOS. The rat epidermal thickness, collagen formation, and positive cell areas of TNF-α, IL-6, and iNOS were quantitatively analyzed using ImageJ software (ImageJ 1.53c).

### 4.15. Statistical Analysis

GraphPad Prism 9 software (GraphPad Prism 9.5.0.730) was applied for data analysis in this study. Independent-sample *t*-test was employed for comparison between two groups, while one-way analysis of variance (ANOVA) was used to analyze the statistical significance among multiple groups.

## Data Availability

The original contributions presented in the study are included in the article/Supplementary Material, further inquiries can be directed to the corresponding author.

## Supplementary Materials

The following supporting information can be downloaded at: https://www.mdpi.com/article/10.3390/gels10100621/s1, Figure S1: TEM images of Nap-F3K hydrogel scale bar = 200 nm., Figure S2: The UV absorption spectrum of DPPH radicals (a) and ABTS radicals (b) during co-incubation with Nap-F3K-CA hydrogel. The UV absorption spectrum of DPPH radicals (c) and ABTS radicals (d) during co-incubation with Nap-F3K hydrogel.
