# Supplementary material for: A Paintable Small-Molecule Hydrogel with Antimicrobial and ROS Scavenging Activities for Burn Wound Healing
Source: Gels. 2024 Sep 26;10(10):621. doi: 10.3390/gels10100621 (PMC11507430; doi:10.3390/gels10100621)
Supplement: Supplementary file 1 [file gels-10-00621-s001.zip › gels-3185140-supplementary.pdf]

gels

Supplementary Materials

A Paintable Small-Molecule Hydrogel with Antimicrobial and ROS Scavenging Activities for Burn Wound Healing

Qingchun Ji †, Kehan Chen †, Han Yi, Bingfang He and Tianyue Jiang *

School of Pharmaceutical Sciences, Nanjing Tech University, Nanjing 211816, China;
qingchunji@njtech.edu.cn (Q.J.); 202261109009@njtech.edu.cn (K.C.); 202361109009@njtech.edu.cn (H.Y.);
bingfanghe@njtech.edu.cn (B.H.)

* Correspondence: tjiang@njtech.edu.cn

† These authors contributed equally to this work.

Supplementary Figures:

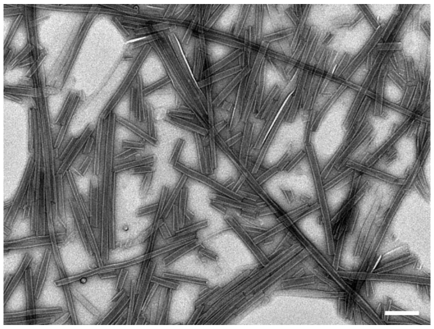

Figure S1. TEM images of Nap-F3K hydrogel scale bar = 200 nm.

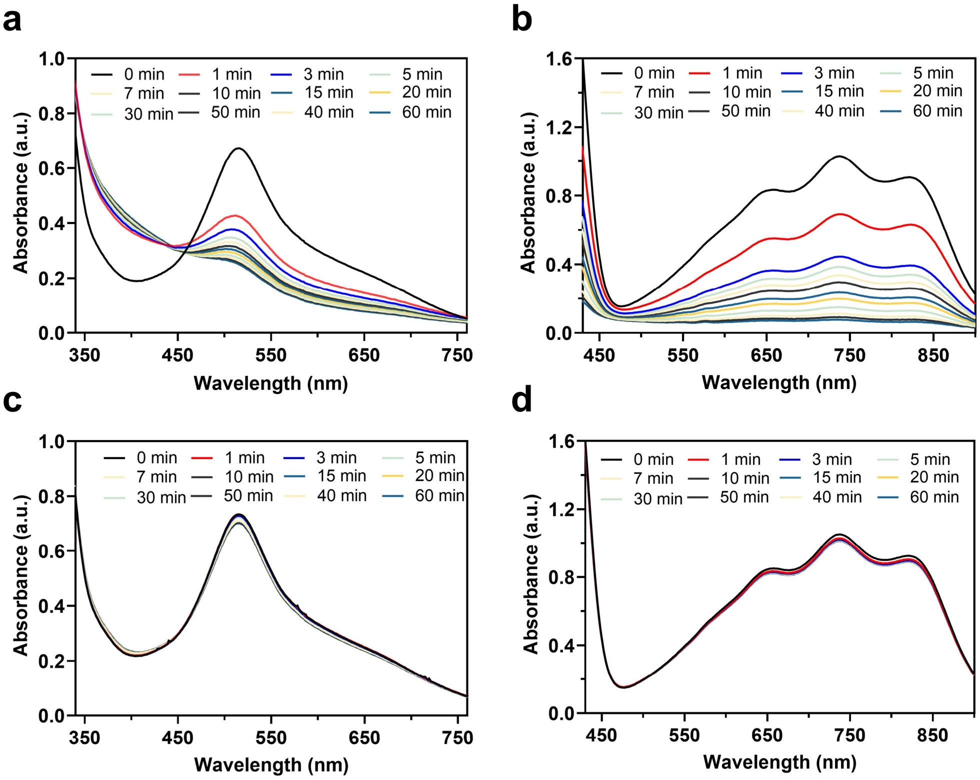

Figure S2. The UV absorption spectrum of DPPH radicals (**a**) and ABTS radicals (**b**) during co-incubation with Nap-F3K-CA hydrogel. The UV absorption spectrum of DPPH radicals (**c**) and ABTS radicals (**d**) during co-incubation with Nap-F3K hydrogel.
